# Supplementary material for: Cryo-EM structure of the human Asc-1 transporter complex
Source: Nat Commun. 2024 Apr 8;15:3036. doi: 10.1038/s41467-024-47468-1 (PMC11001984; doi:10.1038/s41467-024-47468-1)
Supplement: Supplementary file 1 — Supplementary Information [file 41467_2024_47468_MOESM1_ESM.pdf]

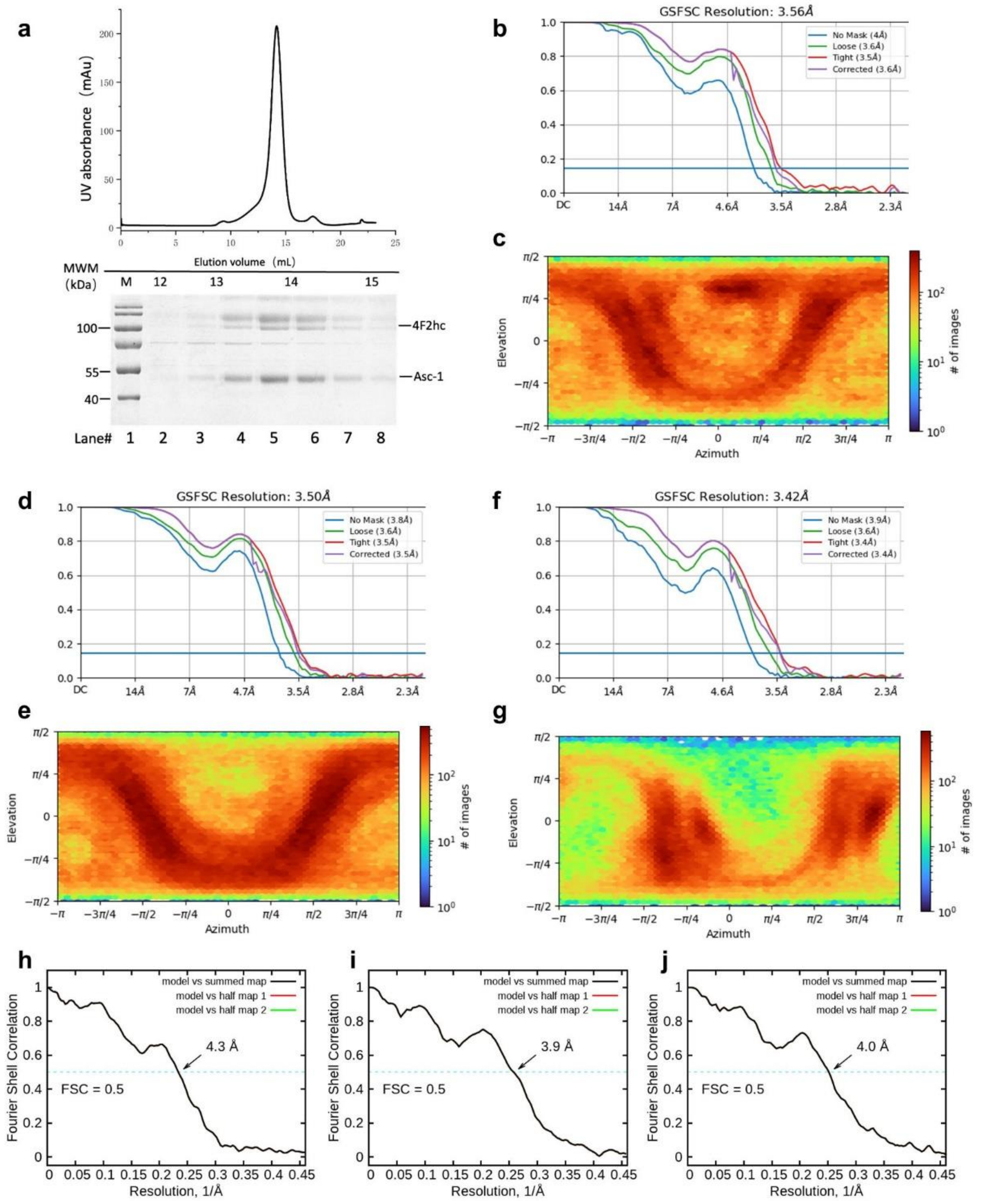

Supplementary Figure 1 Cryo-EM analysis of Asc-1-4F2hc complex.

a, Representative SEC purification of the Asc-1-4F2hc complex. SDS–PAGE was visualized by Coomassie blue staining. **b,** Gold standard FSC curve of the cryoSPARC 3D reconstruction of the map of Apo Asc-1-4F2hc complex. **c,** Euler angle distribution of Apo Asc-1-4F2hc complex. **d,** Gold standard FSC curve of the cryoSPARC 3D reconstruction of the map of Asc-1-4F2hc in complex with D-Ser. **e,** Euler angle distribution of Asc-1-4F2hc in complex with D-Ser. **f,** Gold standard FSC curve of the cryoSPARC 3D reconstruction of the map of Asc-1-4F2hc in complex with L-Ala. **g,** Euler angle distribution of Asc-1-4F2hc in complex with L-Ala. **h-j,** FSC curve of the

refined model of the apo Asc-1-4F2hc **(h)**, Asc-1-4F2hc in complex with D-Ser **(i)** or L-Ala **(j)** versus the overall structure that it is refined against (black); of the model refined against the first half map versus the same map (red); and of the model refined against the first half map versus the second half map (green). The small difference between the red and green curves indicates that the refinement of the atomic coordinates did not suffer from overfitting.

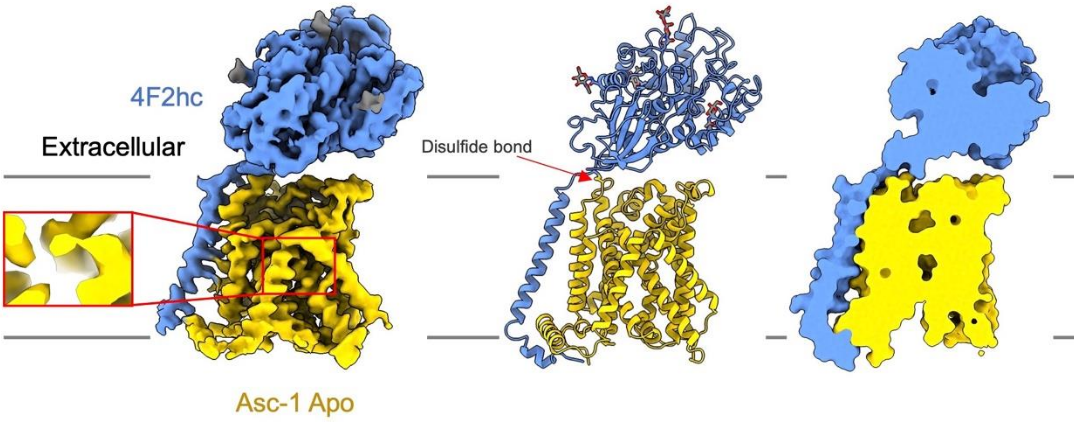

Supplementary Figure 2 Overall structure of Asc-1-4F2hc complex in the apo state
Left: Cryo-EM map of Asc-1-4F2hc in the apo state. The inset shows the density of the empty substrate binding pocket. ***Middle:*** Cartoon structure of the complex. The glycosylation moieties are shown as sticks. ***Right:*** Apo Asc-1 adopts an inward open conformation.

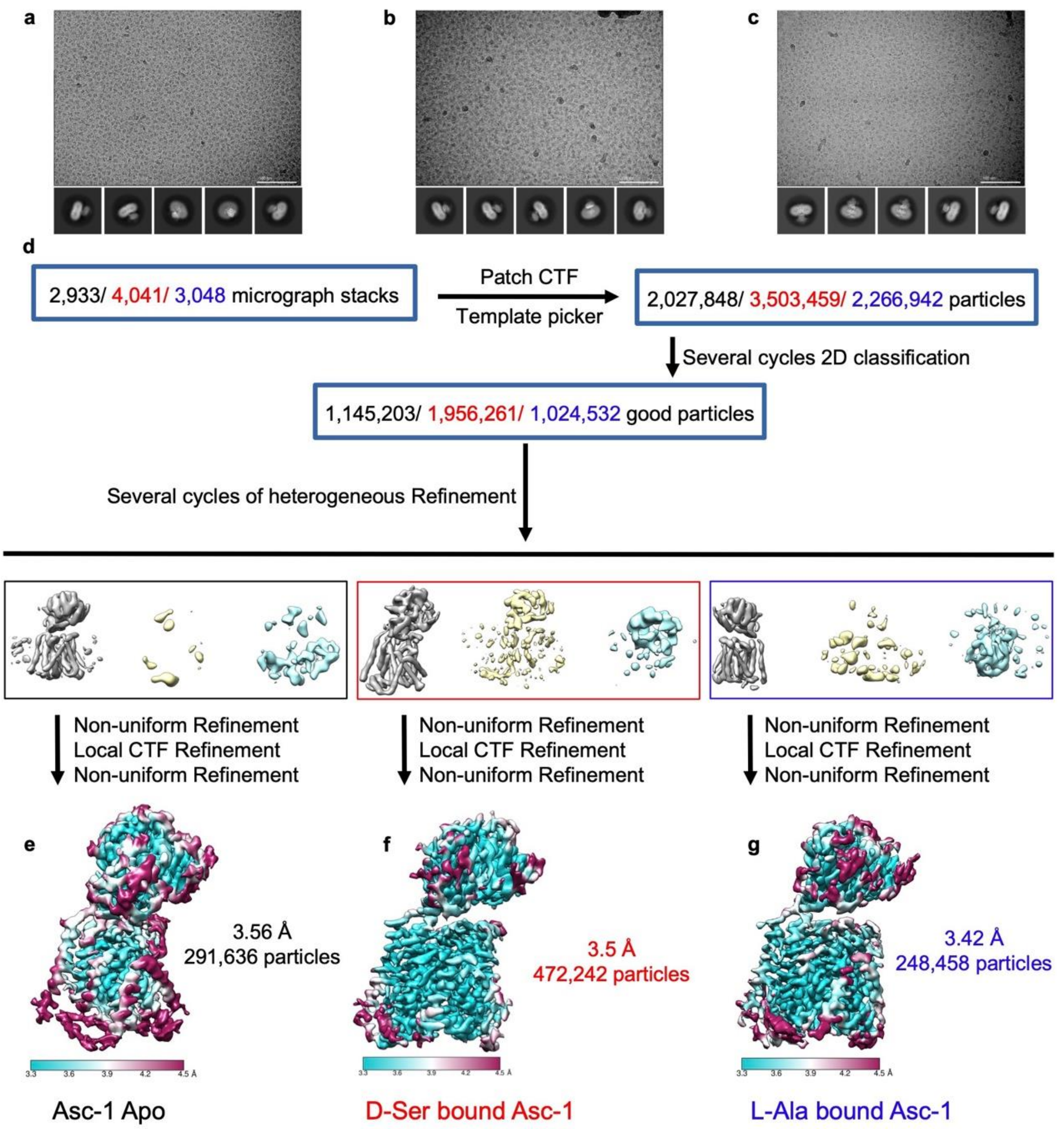

Supplementary Figure 3 Flowchart of cryo-EM data processing.

a-c, Representative micrographs and 2D class averages of apo Asc-1-4F2hc, Asc-1-4F2hc in complex with D-Ser or L-Ala. **d,** Flowchart for cryo-EM data processing. Please refer to the 'Data Processing' in Methods section for details. **e-g,** Local resolution map for the 3D reconstruction of apo Asc-1-4F2hc, Asc-1-4F2hc in complex with D-Ser or L-Ala.

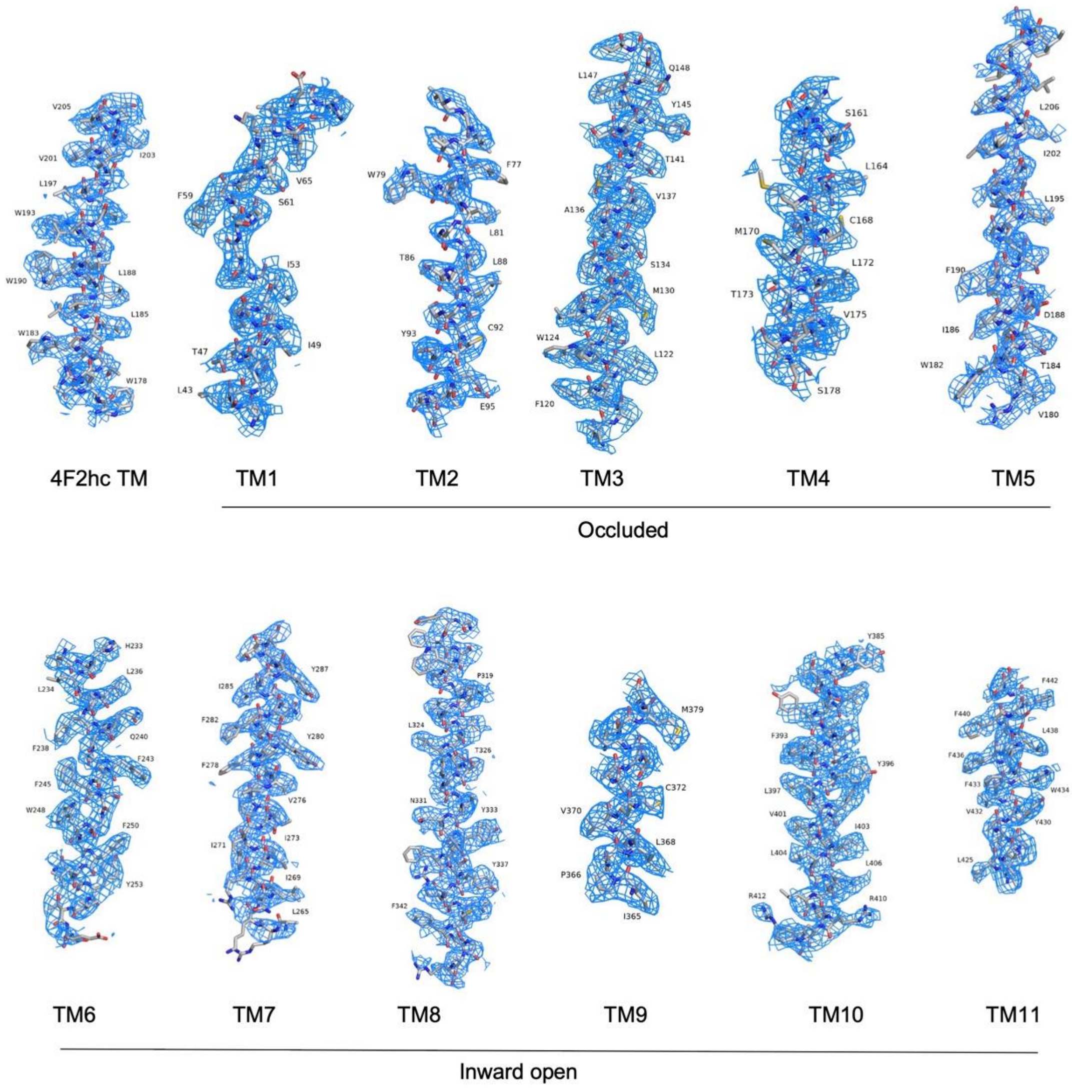

Supplementary Figure 4 Representative cryo-EM density maps of 4F2hc and Asc-1.

Cryo-EM density maps of the transmembrane domain of 4F2hc and Asc-1 are shown at threshold of 7 σ.

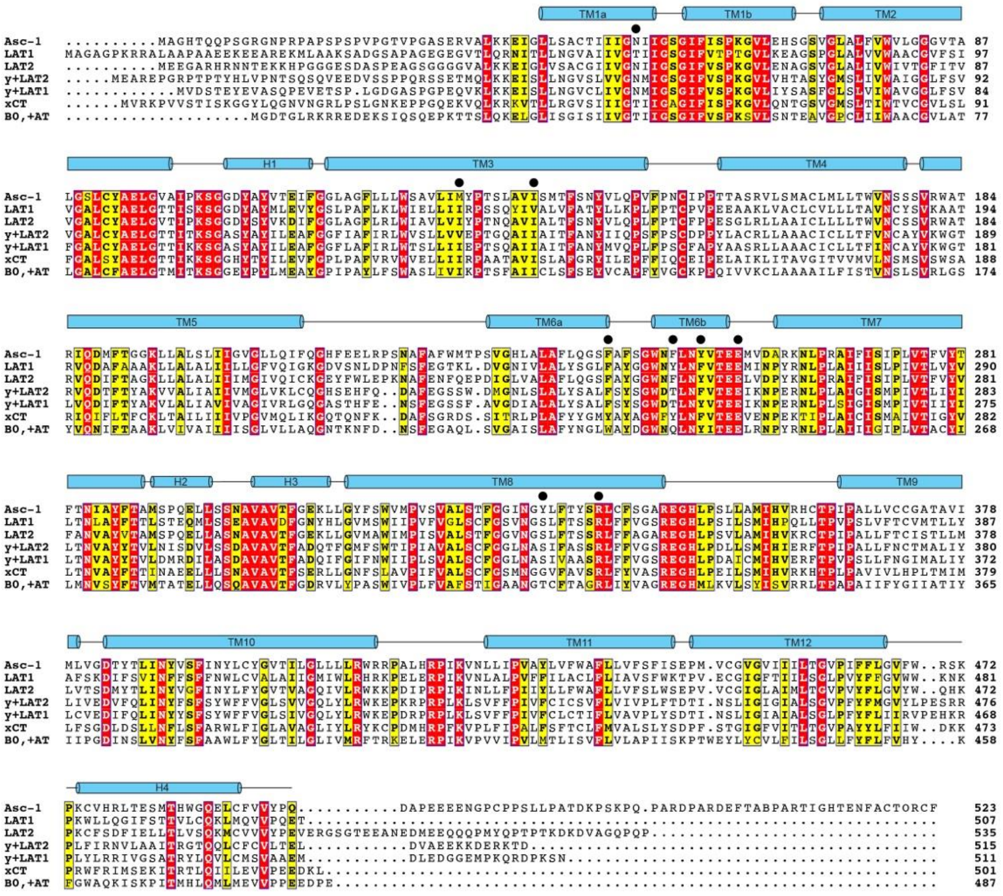

Supplementary Figure 5 Sequence alignment of Asc-1 homologues.

The sequences were aligned using clustalW. The seven aligned sequences are Asc-1, LAT1, LAT2, y^+LAT2, y^+LAT1, xCT, $b^{0,+}$AT from *Homo sapiens*. Amino acids that are identical or conserved in at least six sequences are coloured red or yellow, respectively. The secondary structural elements of Asc-1 are indicated above the sequence alignment. Residues that line the transport path are labelled with a solid black sphere. The UNIPROT IDs of aligned sequences are listed as below. ASC1: Q9NS82; LAT1: Q01650; LAT2: Q9UHI5; y^+LAT2: Q92536; y^+LAT1: Q9UM01; xCT: Q9UPY5; $b^{0,+}$AT: P82251.

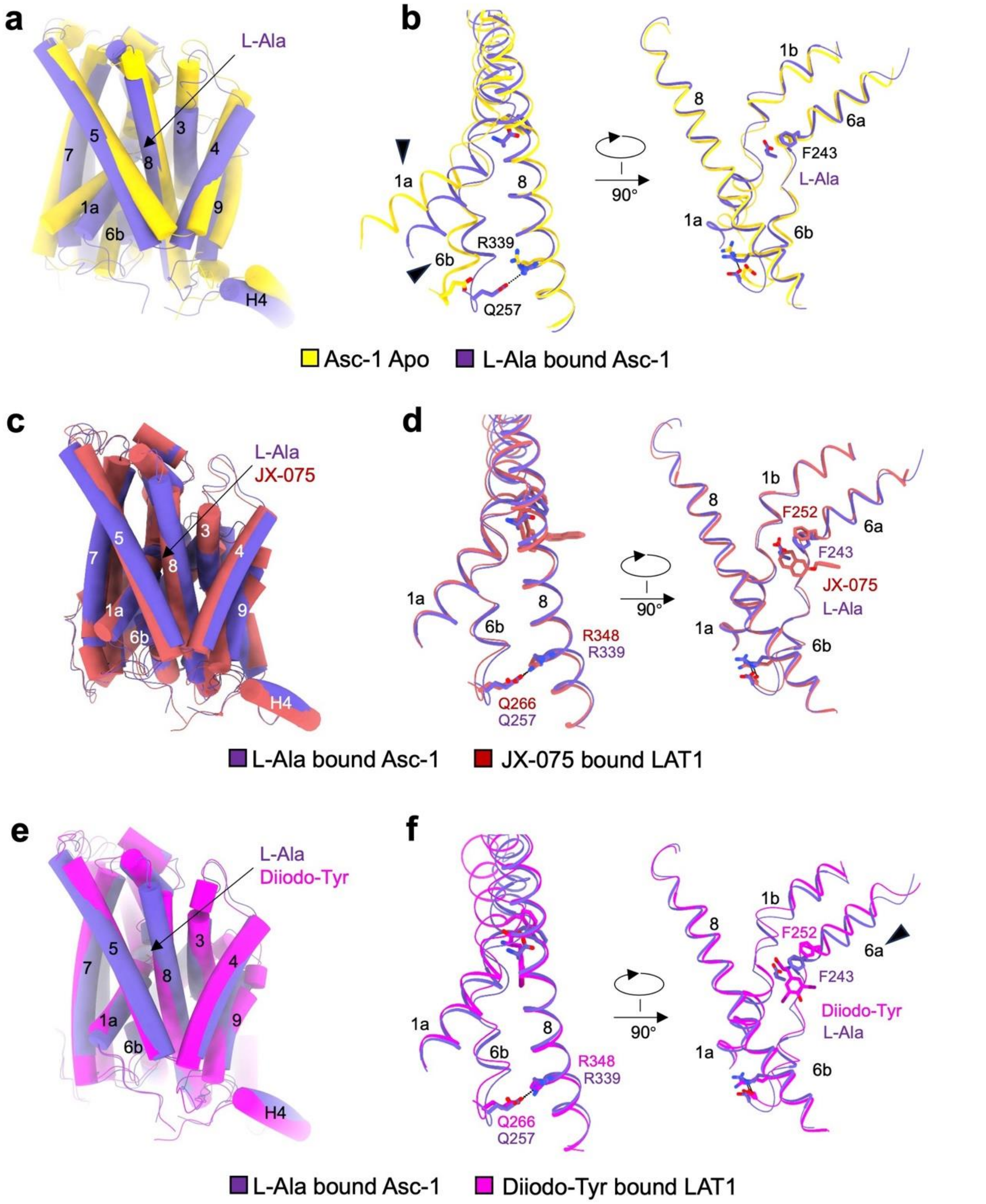

Supplementary Figure 6 The conformational changes of Asc-1 during the transport cycle.

a, b Structure comparison shows that Asc-1 Apo and Asc-1 in complex with L-Ala adopt different conformations. TM1a and TM6b are notably different. The occluded conformation is stabilized by a salt bridge between Glu257 and Arg339. **c, d** Structure comparison of Asc-1 in complex with L-Ala and LAT1 in complex with JX-075 (PDB ID: 7DSK) shows almost same conformation. **e, f** Structure comparison of Asc-1 in complex with L-Ala and LAT1 in complex with Diiodo-Tyr (PDB ID: 7DSQ) shows a

slight difference of TM6a. Asc-1 apo adopts an inward open conformation**.** Asc-1 in complex with L-Ala and LAT1 in complex with JX-075 or Diiodo-Tyr adopt an occluded conformation.

Supplementary Table S1. Cryo-EM data collection, refinement and validation statistics

**Data collection**			
EM equipment		Titan Krios (Thermo Fisher Scientific)	
Voltage (kV)		300	
Detector		Gatan K3 Summit	
Energy filter		Gatan GIF Quantum, 20 eV slit	
Pixel size (Å)	1.087	1.095	1.087
Electron dose (e-/Å2)		50	
Defocus range (µm)		-1.4 ~ -1.8	
Sample	Asc-1-4F2hc apo	Asc-1-4F2hc+D-Ser	Asc-1-4F2hc+L-Ala
Number of collected micrographs	2,933	4,041	3,048
**3D Reconstruction**			
Software	CryoSPARC	CryoSPARC	CryoSPARC
Number of used particles	291,636	472,242	248,458
Resolution (Å)	3.56	3.5	3.42
Symmetry	C1	C1	C1
Map sharpening B-factor (Å2)		-90	
**Refinement**			
Software		Phenix	
Model composition			
Protein residues	924	924	924
Side chains assigned	924	924	924
Sugar	5	5	5
Substrate	0	1	1
D-Ser	0	1	0
L-Ala	0	0	1
R.m.s deviations			
Bonds length (Å)	0.005	0.003	0.003
Bonds Angle (°)	0.731	0.568	0.637
Ramachandran plot statistics (%)			
Preferred	95.43	97.39	94.29
Allowed	4.57	2.61	5.50
Outlier	0.00	0.00	0.22

Original gel image

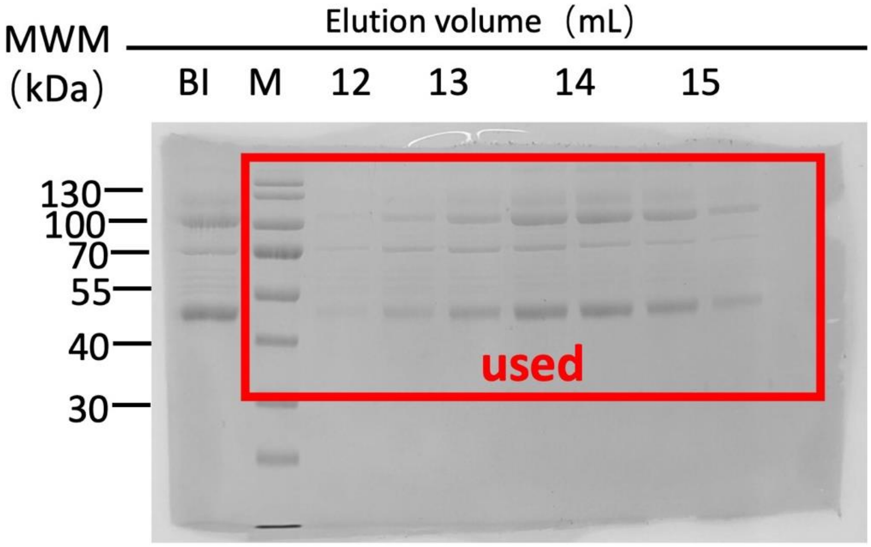

*This gel was shown in Supplementary Fig. S1a
